# The Cincinnati Dental Society

**Published:** 1863-02

**Authors:** 


### Proceedings of Societies.

THE CINCINNATI DENTAL SOCIETY.

By appointment made at a previous informal meeting, this Association met, in the lecture room of the Ohio Dental College, February 3d, 1863, at 7 o'clock P. M.

President Cameron in the chair.

Present, Drs. H. R. Smith, H. A. Smith, B. D. Wheeler, Geo. F. Foote, W. C. Duncan, C. H. James, P. Knowlton, J. Taft, J. G. Cameron.

Essays being called for, Dr. Foote read a paper "*On the influence upon the teeth of breathing through the mouth.*" (Published on another page of this number.) The views set forth elicited considerable discussion.

Dr. Foote. Have seen many cases where breathing was performed through the mouth, and especially during sleep, and in most of them, especially young persons, there was a considerable amount, of that deposit, ordinarily denominated green or black stain, which is so destructive to the teeth; thinks it exists far more frequently, and more abundantly, when breathing is done to any extent through the mouth.

Dr. Knowlton. Thinks we have far too much theory, and not enough facts. The Essay deals too much in the hypothetical; thinks the position taken can not be sustained; thinks that breathing through the mouth does not injure the teeth, but that good pure air would be rather beneficial than otherwise; thinks that the Creator would not make an organ and place it in such an exposed condition, that it would be destroyed or injured, by mere contact with the air. The teeth were designed to be exposed, must necessarily be exposed when speaking; referred to the fact of the teeth so frequently suffering during sickness, and that at such times the mouth is usually open much, but it does not follow that the air does the mischief, that it is usually a want of care in respect to cleanliness, and a vitiation of the secretions, and preponderance of mucus, this will produce decay. Animal matter, flesh for instance, will decompose in a very short time, it is said in two hours, when the mouth is at rest, or the saliva not flowing; an acid is thus formed that is very destructive to the teeth. Our mode of living has much to do with decay of the teeth. Those who live upon plain flood, and observe correct modes of living, have the best teeth. Discussed at some length the materials that enter into the various foods, especially with reference to their bone making properties, he referred to the fact, that in the preparation of some kinds of food the bone making material is removed. Those who most perfectly observe diatetic regulations have the best health.

Dr. Foote. The decomposition of animal substances out of the mouth will not produce or cause decay of the teeth though exposed to its contact for many days; thinks that no injurious acid will be formed by such decomposition within the mouth.

Dr. H. A. Smith. In the decomposition of animal substances, acids may be and often are formed, that will act upon the teeth.

Dr. Foote. Any substance pressing hard between the teeth and infringing upon the gum, will induce an extra exudation of mucus at that point, which failing to be neutralized by the saliva, acts injuriously upon the dentine, inducing decay at the necks of the teeth upon their proximal surfaces.

Dr. H. R. Smith. Thinks the gentlemen have got off of the subject. The Essay has too much theory and not enough facts; we want facts rather than theory. If it is so that children keep their mouths open, what shall we do about it? Can we by a mere *dictum* "shut your mouths" work a revolution in this respect. It is more important and interesting to us to know what to do to conquer disease and to arrest decay, than to discuss vague theories. Thinks the normal mucus is not destructive to the teeth, and that the air is rather preservative than destructive.

Dr. TAFT. The Essay opens rather a new field for thought and discussion; have arrived at no conclusion in regard to the matter suggested by the paper, only desire to elicit the truth; think it not well to be hasty in condemning a matter without consideration. The paper evidently suggests the enquiry, does breathing through the mouth, either directly or indirectly, injure the teeth. The nose and not the mouth is evidently the organ through which nature designed we should breathe, for the most part at least. Holy Writ would seem to convey this idea, for "God formed man of the dust of the ground, and breathed into his nostrils the breath of life." The mouth, however, serves as an alternate. When the nose becomes obstructed, do not suppose that breathing through the mouth injures the teeth directly, if at all, it is indirect. Can hardly suppose that it will in either way work any injury to the teeth, when there is an ordinary flow of saliva. The mouth and all its parts to be in the best condition, should at all times have present a proper relative amount of saliva and mucus. Whenever there is a disturbance of these proportions, especially by a diminution of the saliva, injury may then follow, perhaps both direct and indirect. During sleep and perfect rest, there is only enough saliva and mucus eliminated to keep the mouth in a properly moist condition. In breathing through the mouth during sleep, for instance, the current of air passing through it produces more or less evaporation, and by this evaporation the saliva rather than the mucus, will be removed, and indeed the water of both, in many cases, is by this means removed, so that the mouth will have the dry and "parched" feeling; the dried and thickened mucus will thus be left upon the teeth, and there is little doubt that in such cases it is injurious. When the mouth is closed and in a moist condition, there is a thorough dissemination of the saliva through all parts of the mouth; such is not the case when the mouth is kept open, at rest, and a current of air passing to and fro through it. The mucus is thrown out by the membrane all through the mouth, and hence comes in contact with the teeth, and the saliva under such circumstances does not neutralize it. There is but little doubt that the undiluted and thickened mucus would facilitate the decomposition of foreign substances between and upon the teeth, if not act directly upon them; the latter, very probable, in many cases. Hence, the conclusion that this is, somewhat, a practical subject.

Dr. H. R. Smith. Thinks that the paper assumes that the direct influence of the atmosphere upon the teeth is injurious.

Dr. Foote. The parotid glands throw out but little if any saliva during rest, and very frequently the pure mucus from inflamed surfaces is acidulous, and of an irritating and even excoriating character, as is shown by many circumstances. In the absence of salvia the current of air passing over the mucous surfaces, evaporates the water of the mucous and inflames the membrane beneath it.

Dr. H. A. Smith. Asks if those persons who sleep with the mouth open, do not ordinarily have some irregularity in the arrangement of the teeth, or in the formation of the jaws; replies were made by some members, inclining to the opinion that such is usually the case.

The Association then adjourned to meet at the same place on Friday the 13th at 7 o'clock.

